# Loss of Retinogeniculate Synaptic Function in the DBA/2J Mouse Model of Glaucoma

**DOI:** 10.1523/ENEURO.0421-22.2022

**Published:** 2022-12-21

**Authors:** Jennie C. Smith, Kevin Yang Zhang, Asia Sladek, Jennifer Thompson, Elizabeth R. Bierlein, Ashish Bhandari, Matthew J. Van Hook

**Affiliations:** 1 Truhlsen Eye Institute, Department of Ophthalmology & Visual Sciences, University of Nebraska Medical Center, Omaha, NE 68198; 2 Department of Pharmacology & Experimental Neuroscience, University of Nebraska Medical Center, Omaha, NE 68198; 3 Department of Cellular & Integrative Physiology, University of Nebraska Medical Center, Omaha, NE 68198

**Keywords:** brimonidine, DBA/2J mice, dorsolateral geniculate nucleus, glaucoma, retinogeniculate synapse, thalamus

## Abstract

Retinal ganglion cell (RGC) axons comprise the optic nerve and carry information to the dorsolateral geniculate nucleus (dLGN), which is then relayed to the cortex for conscious vision. Glaucoma is a blinding neurodegenerative disease that commonly results from intraocular pressure (IOP)-associated injury leading to RGC axonal pathology, disruption of RGC outputs to the brain, and eventual apoptotic loss of RGC somata. The consequences of elevated IOP and glaucomatous pathology on RGC signaling to the dLGN are largely unknown yet are likely to contribute to vision loss. Here, we used anatomic and physiological approaches to study the structure and function of retinogeniculate (RG) synapses in male and female DBA/2J (D2) mice with inherited glaucoma before and after IOP elevation. D2 mice showed progressive loss of anterograde optic tract transport to the dLGN and vGlut2 labeling of RGC axon terminals while patch-clamp measurements of RG synaptic function showed that synaptic transmission was reduced in 9-month and 12-month D2 mice because of the loss of individual RGC axon inputs. TC neuron dendrites had reduced Sholl complexity at 12 months, suggestive of delayed reorganization following reduced synaptic input. There was no detectable change in RGC density in 11- to 12-month D2 retinas, quantified as the number of ganglion cell layer-residing somata immuno-positive for NeuN and immuno-negative for the amacrine marker choline acetyltransferase (ChAT). Thus, observed synaptic defects appear to precede RGC somatic loss. These findings identify glaucoma-associated and IOP-associated deficits in an important subcortical RGC projection target, shedding light on processes linking IOP to vision loss.

## Significance Statement

Glaucoma is the leading cause of irreversible blindness worldwide and is commonly associated with elevated intraocular pressure (IOP), which triggers loss of retinal ganglion cell (RGC) somata and connections in the retina, axons in the optic nerve, and outputs to visual centers of the brain. We show here that elevated IOP in the DBA/2J mouse model of inherited glaucoma leads to an early-stage and progressive dysfunction of RGC output synapses in the dorsolateral geniculate nucleus (dLGN). As the dLGN is critical for sending signals to the cortex for conscious vision, these findings demonstrate how RGC output synapse loss can contribute to vision loss in glaucoma.

## Introduction

Glaucoma is a neurodegenerative disease characterized by sensitivity to intraocular pressure (IOP) and progressive retinal ganglion cell (RGC) degeneration (Calkins, 2012, 2021; Weinreb et al., 2014). The goal of this study was to determine the timing and mechanisms by which IOP leads to loss of RGC output synapses [retinogeniculate (RG) synapses] in the mouse dorsolateral geniculate nucleus (dLGN), a subcortical RGC projection target in the thalamus where convergent RGC synaptic inputs to thalamocortical (TC) relay neurons drive TC neuron action potential output to the cortex for conscious vision.

The mechanisms of visual impairment in glaucoma are commonly viewed through the lens of dysfunction progressing toward late-stage apoptotic loss of RGCs. This process occurs as the result of an IOP-induced and age-induced injury to RGC axons at the optic nerve head (Howell et al., 2007a), where the axons exit the eye, triggering retrograde effects on RGCs and their presynaptic partners in the retina. However, elevated IOP also alters the function of RGC axons distal to the optic nerve head and at downstream visual targets in the brain, indicating that dysfunction in the RGC projection is likely to contribute to glaucomatous vision loss. These deficits include disruption of optic nerve active transport (Crish et al., 2010), metabolism (Kleesattel et al., 2015; Inman and Harun-Or-Rashid, 2017; Harder et al., 2020; Casson et al., 2021), and glia (Cooper et al., 2016) as well as alterations to mitochondria (Jassim et al., 2021), RGC excitatory output synapses (Crish et al., 2010; Smith et al., 2016), and the structure and response properties of neurons residing in visual brain nuclei (Bhandari et al., 2019; Van Hook et al., 2020). Evidence to date indicates that many of these functional changes occur early in the pathologic process. Evidence from primate and human studies indicates that glaucoma leads to dendritic remodeling and neuronal atrophy within the visual thalamus (Gupta and Yücel, 2003; Gupta et al., 2006, 2007, 2009). In the superior colliculus of DBA/2J mice with inherited glaucoma, ultrastructural studies show that glaucoma leads to atrophy of presynaptic RGC axon terminals, reduced mitochondrial volume, and decreased size of presynaptic active zones (Smith et al., 2016).

The dLGN is a critical subcortical RGC projection target for conscious vision. In rodents, it receives inputs from ∼40% of RGCs compared with ∼90% of RGCs that project to the superior colliculus (Martin, 1986; Ellis et al., 2016; Seabrook et al., 2017). This contrasts with primates, where RG projections represent a larger proportion of RGC outputs compared with less numerous retinocollicular projections (Perry and Cowey, 1984; Perry et al., 1984). Despite the differences in RG projections between higher-order and lower-order mammals (Kerschensteiner and Guido, 2017; Seabrook et al., 2017; Guido, 2018), the rodent dLGN is an accessible system to shed light on the mechanisms of visual system dysfunction in glaucoma. While we have previously probed early-stage changes to RG function in an inducible mouse ocular hypertension model (Bhandari et al., 2019); here, we sought to map the timing of functional RG synapse loss and impact of IOP across a wider range of time points in chronic glaucoma. In this study, we therefore made use of the DBA/2J mouse (John et al., 1998; Anderson et al., 2002; Libby et al., 2005), a commonly used model system that recapitulates many features of human glaucoma. We find that 9- to 12-month-old DBA/2J mice show IOP-dependent deficits in RGC axonal function, progressive loss of vGlut2-labeled RGC axon terminals, and loss of functional synaptic inputs to each TC neuron. This is accompanied by late-stage reorganization of TC neuron dendrites. Notably, these deficits occurred before detectable RGC somatic loss, as assessed using immunofluorescence staining in retinal flat mounts. Thus, we establish the functional consequences of elevated IOP that impair the conveyance of visual signals from RGCs to their postsynaptic targets in the dLGN. The loss of functional RGC output synapses is a major feature of neurodegenerative disease progression and likely to contribute to glaucomatous vision loss.

## Materials and Methods

### Animals

Animal protocols were approved by the Institutional Animal Care and Use Committee at the University of Nebraska Medical Center. Male and female DBA/2J (D2, The Jackson Laboratory #000671, RRID:IMSR_JAX:000671) and DBA/2J-gpnmb+ (D2-control, The Jackson Laboratory #007048, RRID:IMSR_JAX:007048; Anderson et al., 2002; Howell et al., 2007b) were bred in-house and housed on a 12/12 h light/dark cycle with standard food and water. Intraocular pressure (IOP) was measured approximately monthly beginning at approximately two months of age using an iCare Tonolab rebound tonometer (iCare) in mice that were lightly anesthetized with isoflurane. Measurements were taken within 3 min of isoflurane anesthesia to minimize effects of the anesthesia on IOP. Mice were killed by inhalation of CO_2_ followed by cervical dislocation, in keeping with American Veterinary Medical Association guidelines on euthanasia.

### Cholera toxin B (CTb) injections and analysis

To test for deficits in anterograde transport along the optic tract, mice received a unilateral injection of cholera toxin B subunit coupled to Alexa Fluor 594 (CTb-594, Invitrogen C34777). Mice were anesthetized with isoflurane and treated with proparacaine ophthalmic drops (1%). A Hamilton syringe and 33-gauge needle were used to deliver a unilateral intravitreal injection of ∼1–2 μl of CTb-594 (1 μg/ml). Three to 4 d postinjection, mice were killed with CO_2_ asphyxiation and cervical dislocation. Brains were dissected, rinsed briefly in PBS, and fixed by immersion in 4% paraformaldehyde (PFA) in PBS overnight. After fixation, brains were rinsed in PBS, cryoprotected overnight in 30% sucrose, embedded in 3% agar, and sliced into 100-μm-thick slices on a Leica VT1000S vibratome. Every other section containing the dLGN was mounted on SuperFrost Plus slides (Fisher Scientific) and coverslipped with Vectashield Hardset (Vector). CTb-594 images of the contralateral dLGN were acquired using a 10× objective lens on an Olympus BX51WI microscope with a Tucsen monochrome camera. To analyze CTb-594 labeling, each image was thresholded in ImageJ based on a region outside of the dLGN and the number of CTb-594 pixels was counted using the histogram. In this way, the total volume of the dLGN labeled by CTb-594 was calculated in serial dLGN sections for each mouse.

### Immunofluorescence staining

Retinal ganglion cell axon terminals were labeled by immunofluorescence staining for vGlut2. After euthanasia, brains were dissected into PBS and fixed for 4 h in 4% PFA. Brains were then rinsed in PBS, cryoprotected overnight in 30% sucrose, embedded in 3% agar, and cut into 50-μm-thick sections using a Leica VT1000S vibratome. Sections were mounted on SuperFrost Plus slides. For staining, sections were rinsed in PBS, blocked/permeabilized with 0.5% Triton X-100, 5.5% goat serum and 5.5% donkey serum and incubated overnight with a guinea pig polyclonal vGlut2 antibody (1:250, Millipore AB2251-1, RRID:AB_2665454). vGlut2 is a relatively specific marker of RGC axon terminals in the dLGN (Fujiyama et al., 2003; Land et al., 2004; Yoshida et al., 2009; Koch et al., 2011; Seabrook et al., 2013; Bhandari et al., 2022). After primary antibody incubation, slices were rinsed 6 × 10 min and incubated with an Alexa Fluor 488-conjugated goat-anti-guinea pig IgG (1:200, Invitrogen A-11073, RRID:AB_2534117) for 4 h, washed 3 × 5 min in PBS, and coverslipped with Vectashield Hardset. vGlut2 fluorescence was imaged on a Scientifica two-photon microscope with a MaiTai HP Ti:sapphire laser tuned to 800 nm with a 370 × 370 μm field of view (2.77 pixels/μm) centered on the dLGN core. The signal in a single optical section was automatically thresholded and vGlut2 puncta with a size threshold of 6 μm^2^ were detected using the Synapse Counter plug-in in ImageJ (Dzyubenko et al., 2016).

For measuring retinal ganglion cell density, retinas from D2 and D2-control mice (11–12 months of age) were dissected free from the eyecup in Ames solution (US Biologicals, A13722525L). Relieving cuts were made and retinas were mounted on nitrocellulose membranes, after which they were fixed in 4% PFA for 30 min. After 3 × 5 min washes in PBS, retinas were blocked/permeabilized using a solution containing 1% Triton X-100, 5.5% donkey serum, 5.5% goat serum, and 0.5% dimethylsulfoxide for 1 h. Following blocking and permeabilization, retinas were incubated overnight at 4°C in the same solution containing a rabbit-anti-choline acetyltransferase (ChAT) monoclonal antibody (1:1000, Abcam ab178850, RRID:AB_2721842) and a guinea pig-anti-NeuN polyclonal antibody (1:500, Millipore ABN90, RRID:AB_11205592). After 6 × 10 min of washing in PBS, retinas were incubated in Alexa Fluor-conjugated secondary antibodies (1:200 goat-anti-guinea pig IgG 568, Invitrogen A-11075, RRID:AB_141954; 1:200 donkey-anti-rabbit IgG-488, Invitrogen A-21206, RRID:AB_2535792) for 4 h, washed 3 × 5 min, mounted on SuperFrost Plus slides and coverslipped with Vectashield Hardset. NeuN and ChAT-labeled cells in the ganglion cell layer were imaged on a two-photon microscope in three to four quadrants of the central retina (∼500 μm from the optic nerve head) and peripheral retina (∼1700 μm from the optic nerve head). RGCs counts were performed using an ImageJ macro in which the maximum intensity projections were thresholded, despeckled, and inverted followed by application of the “dilate,” “fill holes,” and “watershed” commands. Finally, the Analyze Particles tool was used to detect objects with a circularity of 0.3–1 with a size threshold of 30 μm^2^. Separately, ChAT-labeled amacrine cells were counted with a circularity of 0.4–1 and a size threshold of 80 μm^2^. In a subset of images (*n* = 9), we found that 66% of ChAT^+^ cells detected using these parameters were also detected as NeuN^+^. Thus, the RGC number was taken as the difference of NeuN-labeled cells and NeuN/ChAT double-labeled cells, similar to the approach described previously (Buckingham et al., 2008). Density was analyzed separately in central and peripheral retina using the mean cell counts analyzed in this manner from the three to four central or peripheral images for each eye.

### Patch-clamp electrophysiology

For measurements of retinogeniculate synaptic function, parasagittal sections containing the dLGN (J.P. Turner and Salt, 1998; C. Chen and Regehr, 2000) were prepared using the “protected recovery” method (Ting et al., 2014, 2018) that involved sectioning in an ice-cold artificial CSF (aCSF; 128 NaCl, 2.5 KCl, 1.25 NaH_2_PO_4_, 24 NaHCO_3_, 12.5 glucose, 2 CaCl_2_, and 2 MgSO_4_ and continuously bubbled with a mixture of 5% CO_2_ and 95% O_2_) followed by a 12-min incubation in an *N*-methyl-D-glucamine-based solution (in mm: 92 NMDG, 2.5 KCl, 1.25 NaH_2_PO_4_, 25 glucose, 30 NaHCO_3_, 20 HEPES, 0.5 CaCl_2_, 10 MgSO_4_, 2 thiourea, 5 L-ascorbic acid, and 3 Na-pyruvate, warmed to 33°C). After an additional >1 h recovery in aCSF at room temperature, slices were transferred to a recording chamber on an Olympus BX51-WI upright microscope and superfused with aCSF supplemented with 60 μm picrotoxin at ∼2 ml/min bubbled with 5% CO_2_ and 95% O_2_ and warmed to 30–33°C with an in-line solution heater (Warner Instruments).

Thalamocortical (TC) relay neurons were targeted for whole-cell voltage clamp recording based on soma size and morphology using a pipette solution comprised of (in mm) 120 Cs-methanesulfonate, 2 EGTA, 10 HEPES, 8 TEA-Cl, 5 ATP-Mg, 0.5 GTP-Na_2_, 5 phosphocreatine-Na_2_, 2 QX-314 (pH 7.4, 300 mOsm). Electrophysiology was performed using a Multiclamp 700B or 700A amplifier, a Digidata 1550B digitizer, and pClamp 10 or 11 software (Axon/Molecular Devices). The holding voltage was −70 mV after correction for the liquid junction potential, which was measured as 10 mV. Series resistance was partially compensated (65–75%) during voltage-clamp recordings of evoked EPSCs, but not during recordings of spontaneous EPSCs (sEPSCs). Signals were sampled at 10 kHz and filtered at 1.6 kHz during acquisition. A concentric bipolar stimulating electrode was positioned in the optic tract anterior and ventral to the ventral LGN (∼1–1.5 mm from the dLGN) and used to deliver pairs of current stimuli to RGC axons from an AM Systems Model 2100 Isolated Pulse Stimulator (0.3–0.5 ms, 200-ms interstimulus interval).

To measure the maximal AMPA-receptor-mediated EPSCs (EPSC_AMPA_), representing the response evoked by all intact RGC inputs converging onto a given TC neuron (Hooks and Chen, 2006, 2008; Litvina and Chen, 2017a), we increased stimulus intensity until the response amplitude plateaued. This was sometimes up to 10 mA stimulus amplitude, which was the maximum of our stimulus generator and only used to ensure the maximum number of inputs were activated; we did not observe signs of damage to the slice after brief use of this stimulus intensity, as we were able to reduce the stimulus amplitude to lower levels and continue to obtain robust responses. NMDA-receptor EPSCs (EPSC_NMDA_) were measured as the amplitude 15 ms poststimulus while the TC neurons were voltage clamped at +40 mV. At this time point, the inward AMPA current had decayed to <4% of its peak amplitude, consistent with prior work measuring NMDA_EPSC_ at RG synapses (C. Chen and Regehr, 2000; D.J. Lin et al., 2014) and indicating this represents a suitable time point for NMDA_EPSC_ measurement. To measure RGC convergence, the stimulus intensity was reduced until it evoked an EPSC representing the input from a single RGC axon (single-fiber EPSC, EPSCsf), defined as the EPSC amplitude recorded when a given stimulus failed to evoke a response approx. 50% of the time. The “fiber fraction” (Hooks and Chen, 2006, 2008; Litvina and Chen, 2017a), which is an estimate of the single fiber contribution to the maximal EPSC, and thus, a quantifiable metric for measuring RGC input convergence onto postsynaptic TC neurons, was calculated as the ratio of the EPSCmin/EPSC_AMPA_. These electrophysiology data were analyzed with ClampFit 11.

sEPSCs were recorded over 60 s in the absence of stimulation and in the presence of 60 μm picrotoxin. sEPSCs were detected and amplitude and frequency were analyzed using MiniAnalysis software (Synaptosoft) with an amplitude threshold of 4.5 pA. Fits of sEPSC amplitude and baseline noise histograms revealed good separation of sEPSCs from recording noise with these detection parameters. To test whether sEPSCs were dependent on action potential firing in the dLGN slices, after recording control sEPSCs in a handful of slices, they were then were treated with 500 nm tetrodotoxin (TTX; Abcam, ab120054) and sPESCs were recorded again.

### Single-neuron dye fills and dendritic reconstruction

For single-neuron dendritic reconstructions, TC neurons in the dLGN core (Krahe et al., 2011) were targeted for whole-cell patch clamp recording in 250-μm-thick coronal sections prepared using the protected recovery method, as described above. The pipette solution was either the Cs-methanesulfonate solution, as above, or was a K-gluconate solution (in mm, 120 K-gluconate, 8 KCl, 2 EGTA, 10 HEPES, 5 ATP-Mg, 0.5 GTP-Na_2_, 5 Phosphocreatine-Na_2_) supplemented with 2% Neurobiotin (Vector Laboratories, SP-1120) and 10 μm CF568 (Biotium). Neurobiotin and CF568 were injected using square-wave current injections (500 pA peak to trough, 2 Hz) for 10–15 min in current clamp mode, after which slices were fixed in 4% PFA for 1 h and incubated for a week in 10 μg/ml streptavidin-568 in PBS with 1% Triton X-100 at 4°C. After incubation, slices were washed 3 × 10 min in PBS, mounted on Superfrost Plus slides and coverslipped with Vectashield Hardset (Vector Laboratories, H-1400). Filled TC neurons were imaged on a two-photon microscope and dendrites were reconstructed using Simple Neurite Tracer plug-in in ImageJ. Sholl analysis was performed in ImageJ on a two-dimensional projection of the reconstructed dendrites (10 μm spacing between Sholl rings). Equivalent dendritic field diameter was calculated from the area of a convex polygon drawn by connecting TC neuron dendritic tips in ImageJ.

### Experimental design and statistical analyses

Statistical analysis was performed using GraphPad Prism 9. Normality of the data were assessed using a D’Agostino and Pearson test. When data were normally distributed, significance was assessed using a one-way ANOVA with Dunnett’s multiple comparison tests. To avoid pitfalls from pseudoreplication (Eisner, 2021), statistical significance was measured using one-way nested ANOVA with a Dunnett’s multiple comparisons *post hoc* test when we made multiple measurements from single animals (i.e., multiple cells from each mouse in a dataset). Data sets that followed a logarithmic distribution were log transformed before statistical testing. For all statistical tests, *p* < 0.05 was considered statistically significant. Sample sizes (number of mice and the number of cells), statistical tests, *F* or *t* statistics, and *p*-values are reported in the figure legends. For *F* statistics, we report the between-groups and within-groups degrees of freedom: *F*_(dfbetween,dfwithin)_. We also report the degrees of freedom for the *t* statistics: *t*(df). The IOP integral (mmHg*months, averaging the measurements from both eyes) was measured as the area under the curve of the monthly IOP readings and used to test for a relationship of IOP with measures of dLGN synaptic structure and function. Linear regressions show the best-fit line with 95% confidence bands. Data are displayed as individual data points and mean ± SEM or median ± interquartile range (IQR), as indicated in figure legends. Control groups represent a mix of D2-control mice from 6 to 12 months of age, with ages and numbers indicated in the figure legends.

## Results

**Figure 1.**
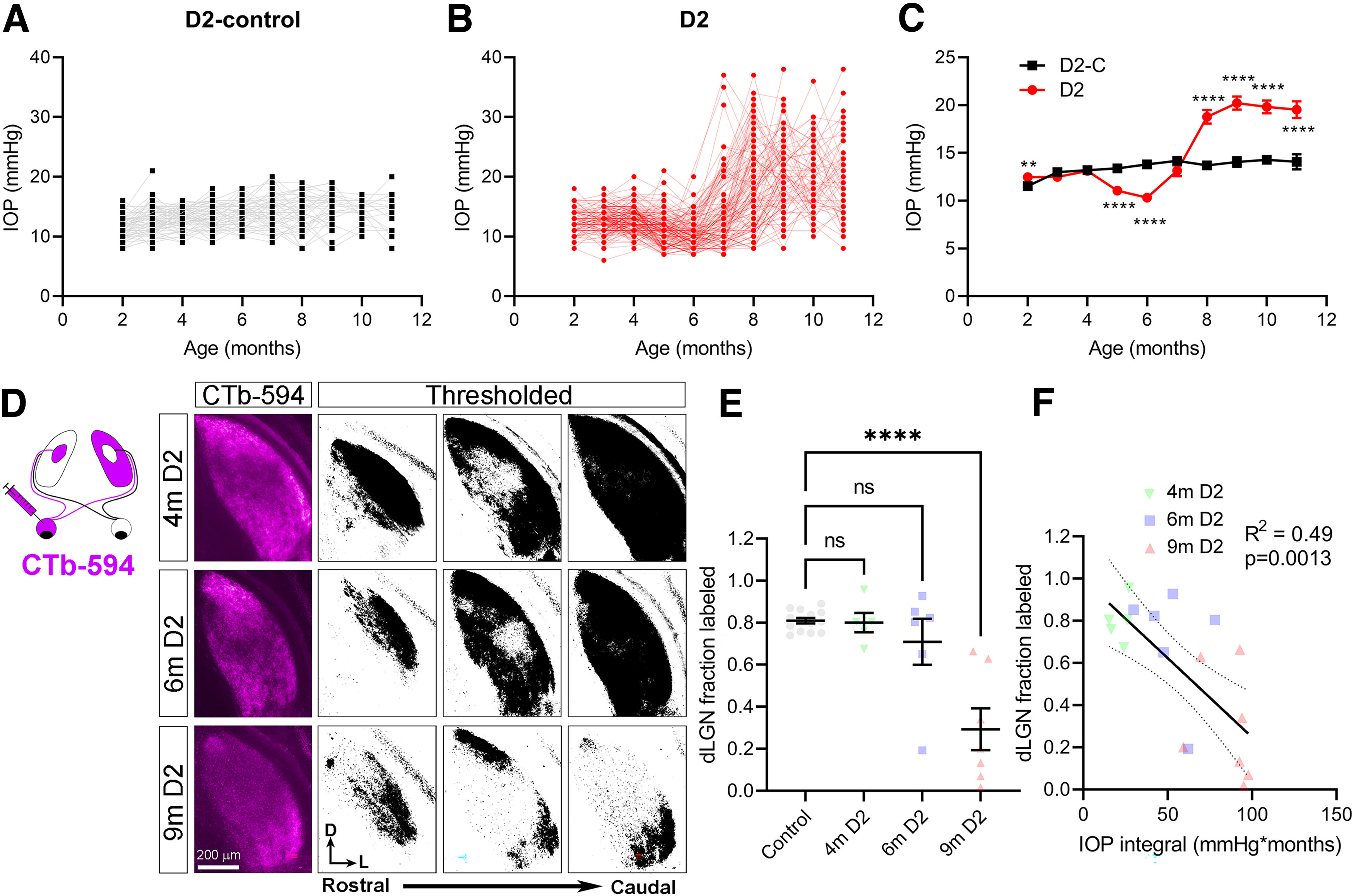
Elevated intraocular pressure and deficits in anterograde transport to the dLGN. ***A***, Intraocular pressure (IOP) measurements from DBA/2J-gpnmb+ (D2-control) mice (*n* = 110 eyes from 55 mice included in this study). ***B***, IOP measurements from DBA/2J (D2) mice (*n* = 168 eyes from 84 mice). ***C***, Mean (±SEM) IOP measurements from D2 and D2-control eyes. Unpaired *t* tests: 2 months (m) *t*_(147)_ = 2.942, *p* = 0.0038; 3m *t*_(205)_ = 1.539, *p* = 0.13; 4m *t*_(183)_ = 0.275, *p* = 0.78; 5m *t*_(203)_ = 6.883, *p* = 1.0 × 10^−10^; 6m *t*_(163)_ = 8.992, *p* = 2.3 × 10^−14^; 7m *t*_(143)_ = 1.47, *p* = 0.14; 8m *t*_(155)_ = 6.144, *p* = 6.6 × 10^−9^; 9m *t*_(119)_ = 7.29, *p* = 4.0 × 10^−11^; 10m *t*_(81)_ = 6.881, *p* = 1.3 × 10^−9^; 11m *t*_(69)_ = 4.63, *p* = 2.5 × 10^−5^. ***D***, Fluorescently-tagged cholera toxin-B (CTb) was injected unilaterally in to the vitreous and the area of labeled contralateral dLGN was measured based on fluorescence signal in serial dLGN sections. ***E***, Group data (mean ± SEM) showing fraction of CTb-labeled dLGN. There was a significant difference among groups (one-way ANOVA, *F*_(3,28)_ = 15.0, *p* = 5.2 × 10^−6^) and the 9m group significantly differed from the control group (Dunnett’s multiple comparison test; *p* < 1 × 10^−15^). ***F***, For the D2 mice, there was a significant negative correlation (linear regression with 95% confidence interval) of the fraction of dLGN labeled by CTb with the IOP integral (Pearson correlation, *F*_(1,16)_ = 15.22, *p* = 0.0013). Sample size: Control *n* = 14 (controls by age: 4m *n* = 4, 6m *n* = 6, 9m *n* = 4); 4m D2 *N* = 5; 6m D2 *n* = 6; 9m D2 *n* = 7. **p < 0.005; ****p < 0.00005.

To determine how IOP elevation in DBA/2J mice influences the dLGN, we performed experiments to longitudinally monitor IOP and assess anterograde axoplasmic transport of fluorescently-tagged cholera toxin B (CTb) subunits (Fig. 1). D2 mice showed an increase in IOP (*n* = 168 eyes, 84 mice) beginning at approximately seven months of age. IOP elevation was variable, as we and others have reported previously (Libby et al., 2005; Inman et al., 2006). Compared with DBA/2J-gpnmb^+^ (D2-control mice, *n* = 110 eyes, 55 mice), IOP was significantly elevated after eight months. Notably, IOP in D2 mice was significantly lower than in D2-controls at five and six months (Fig. 1*B*,*C*), similar to what we have observed previously (Bierlein et al., 2022) and is apparent in figures from other prior studies (Libby et al., 2005; A.J. Turner et al., 2017; Rohowetz et al., 2021). While female DBA/2J mice had, on average, higher IOP than males at ages more than eight months, consistent with prior work (Libby et al., 2005), the male and female IOP measurements overlapped considerably. Using serial histologic sections of the dLGN, we quantified the total fraction of dLGN labeled by anterogradely-transported CTb-594 (Fig. 1*D–F*). Approximately 80% of the dLGN was labeled in D2-control mice, which was similar to the amount labeled in four-month-old D2 mice and was the result of result of complete labeling from contralateral projections and no labeling in the ipsilateral projection region. By nine months of age, 29 ± 1% of the dLGN was labeled, and the pattern appeared to be the result of regional loss of transport, similar to what has been documented for the superior colliculus (Crish et al., 2010). There was also a weak but statistically significant correlation of CTb-594 labeling with the IOP integral (Fig. 1*F*), suggesting a functional link between IOP and deficits in anterograde transport to the dLGN.

**Figure 2.**
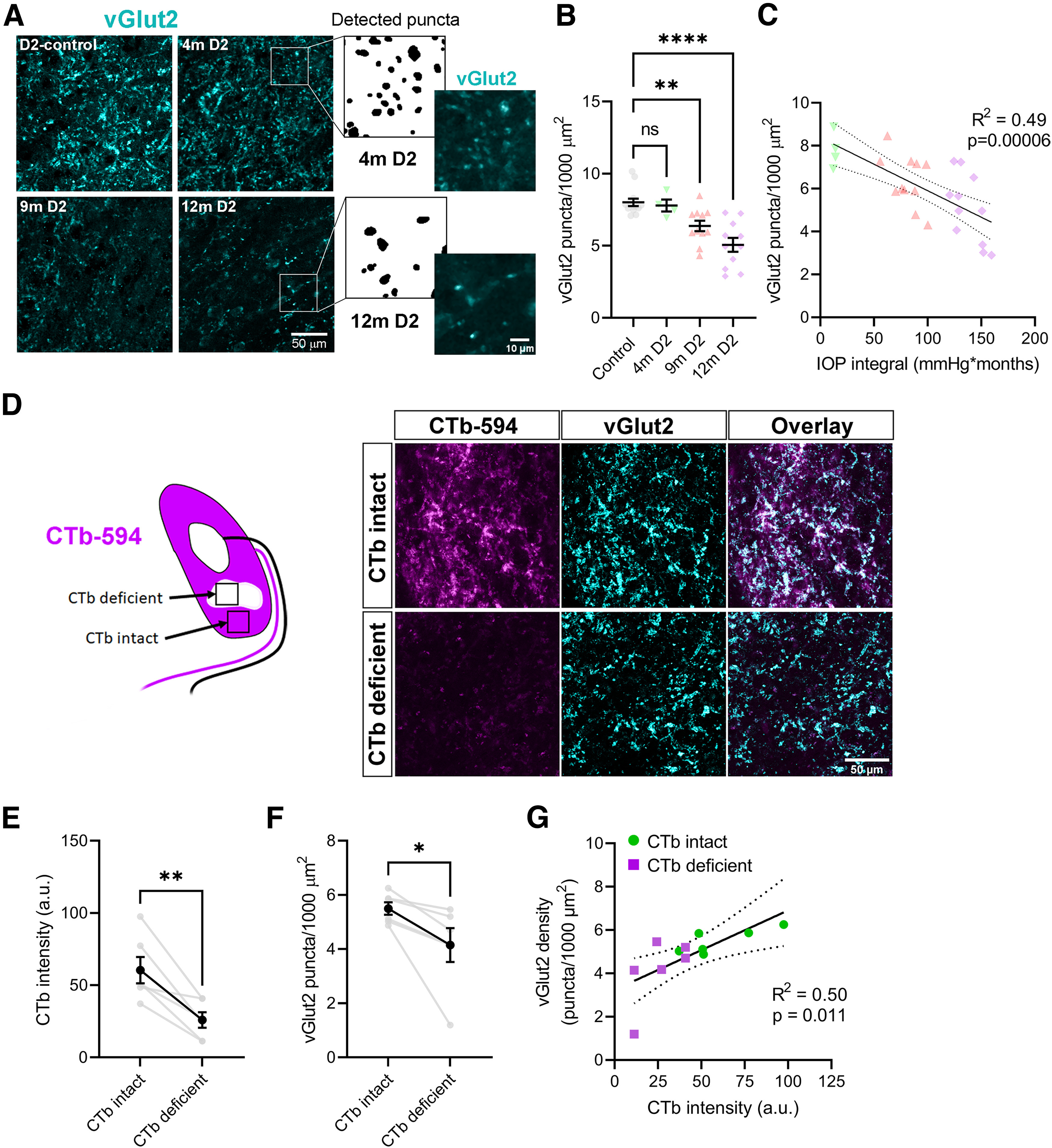
Loss of vGlut2-labeled RGC axon terminals in the dLGN is associated with transport deficits in DBA/2J mice. ***A***, Single optical sections of dLGN labeled with an anti-vGlut2 antibody from a D2-control mouse and D2 mice at 4 months (m), 9m, and 12m of age. Zoomed-in images with detected puncta from the 4m and 12m D2 images are shown to the right. ***B***, Group data (mean ± SEM) showing density of detected vGlut2 puncta. There was a significant difference among groups (one-way ANOVA, *F*_(3,35)_ = 12.87; *p* = 8.0 × 10^−6^) with 9m and 12m groups differing significantly from the control group (Dunnett’s multiple comparison: 4m *p* = 0.98; 9m *p* = 0.0070; 12m *p* < 1 × 10^−15^). ***C***, vGlut2 density was significantly correlated with IOP integral (*F*_(1,24)_ = 23.11; *p* = 0.000068). ***D***, Analysis of vGlut2 density in regions of the dLGN with intact or deficient anterograde transport of unilaterally-injected CTb. D2-control mice total *n* = 13 (D2-control by age: 4m *n* = 6; 9m *n* = 5; 12m *n* = 2); 4m D2 *n* = 4; 9m D2 *n* = 11; 12m D2 *n* = 11. ***E***, Quantification (mean ± SEM) of CTb pixel intensity in “intact” or “deficient” dLGN regions (*t*_(5)_ = 4.489, *p* = 0.0065, paired *t* test). ***F***, Quantification of vGlut2 density (mean ± SEM) in dLGN regions with intact or deficient CTb labeling (*t*_(5)_ = 2.743, *p* = 0.041, paired *t* test, *n* = 6 D2 mice). ***G***, Significant positive correlation (linear regression with 95% confidence interval) of vGlut2 density with intensity of CTb labeling (Pearson correlation, *F*_(1,10)_ = 9.812, *p* = 0.011). *p < 0.05; **p < 0.01; ****p < 0.00005.

To test for loss of RGC axon terminals, we next immunostained dLGN sections for vGlut2 (Fig. 2). In these experiments, the density of vGlut2-labeled puncta was comparable between D2-control and four-month-old D2 mice. In older D2 mice, the density of vGlut2-labeled puncta showed a progressive reduction, being lower in 9-month (m) and lower still in 12m D2 mice. Of note, we observed very dim vGlut2 labeling of presumptive TC neuron somata, which most apparent in sections from 9m and 12m D2 mice with diminished synaptic labeling. This is consistent with prior work indicating that TC neuron axon terminals in the visual cortex express vGlut2 (Nahmani and Erisir, 2005). vGlut2 density in D2 mice was negatively correlated with the IOP integral (Fig. 2*C*). To better understand the relationship between loss of vGlut2 puncta and deficits in anterograde transport to the dLGN, we performed vGlut2 immunofluorescence staining on dLGN sections that had also been labeled via anterograde CTb transport (Fig. 2*D–G*). We compared vGlut2 density in regions of 9m D2 dLGN with intact CTb labeling (“CTb-intact”) with dLGN regions that had little CTb labeling (“CTb-deficient”). In this experiment, we found that CTb-deficient regions of the dLGN also had lower vGlut2 puncta density compared with vGlut2 density in the CTb-intact regions. However, this was not a one-to-one relationship; while there was a correlation of CTb labeling intensity with vGlut2 puncta density, regions with very little CTb still had labeling for vGlut2. This result is consistent with findings from the superior colliculus, where anterograde transport loss precedes synaptic loss (Crish et al., 2010; Smith et al., 2016). However, some remaining vGlut2 signal might also originate from tectogeniculate synaptic inputs arising from vGlut2-expressing projection neurons in the stratum griseum superficiale of the superior colliculus (Fremeau et al., 2001; Bickford et al., 2015) or potentially other “replacement terminals” (Whyland et al., 2022).

**Figure 3.**
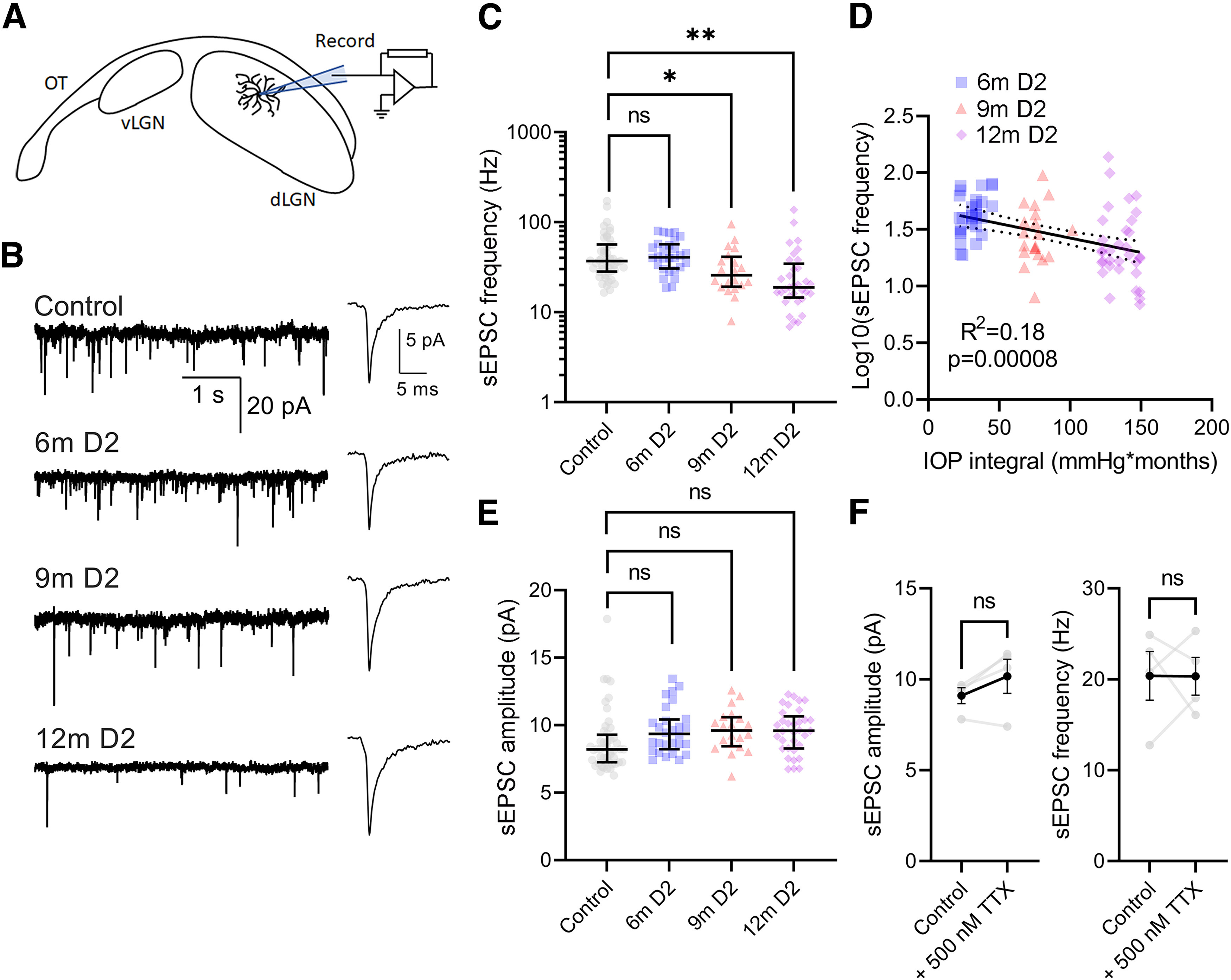
Progressive loss of miniature EPSCs recorded from dLGN thalamocortical relay neurons in DBA/2J mice. ***A***, Recording schematic of optic tract (OT), ventral lateral geniculate nucleus (vLGN), and dLGN with patch clamp electrode in parasagittal slice. ***B***, Left, Example 5-s duration traces of spontaneous quantal EPSCs (sEPSCs) recorded in the without stimulation from D2-control and D2 mice. Right, Average of the detected sEPSC waveforms. ***C***, Group data (median ± IQR) of sEPSC frequency. There was a significant difference among groups (nested one-way ANOVA, *F*_(3,33)_ = 6.038, *p* = 0.0021) and 9-month and 12-month groups differed significantly from control (Dunnett’s multiple comparison: 6m *p* = 0.99; 9m *p* = 0.026; 12m *p* = 0.0078). ***D***, There was a weak but significant correlation of Log10(sEPSC frequency) measured in TC neurons from D2 mice with the IOP integral (*F*_(1,79)_ = 17.2; *p* = 0.000084). ***E***, Group data (median ± IQR) of sEPSC amplitude. There was no significant difference among groups (nested one-way ANOVA, *F*_(3,34)_ = 1.696, *p* = 0.19) and individual groups were not significantly different from the control (Dunnett’s multiple comparison, 6m *p* = 0.24; 9m *p* = 0.29; 12m *p* = 0.21). Group sizes: D2 control total *n* = 48 cells, 13 mice (D2-control by age: 6m *n* = 11 cells, 3 mice; 9m *n* = 27 cells, 8 mice; 12m *n* = 10 cells, 2 mice). 6m D2 *n* = 28 cells, 7 mice; 9m D2 *n* = 20 cells, 8 mice; 12m D2 *n* = 33 cells, 10 mice. ***F***, Group data (mean ± SEM) of sEPSC amplitude and frequency recorded before and after bath application of 500 nm tetrodotoxin (TTX; *n* = 4 TC neuron recordings). TTX did not have a significant effect on either amplitude (*t*_(3)_ = 2.020, *p* = 0.137, paired *t* test) or frequency (*t*_(3)_ = 0.014, *p* = 0.99, paired *t* test). *p < 0.05; **p < 0.01; ns *p* > 0.05.

The above data imply that impaired axonal function (as indicated by reduction in anterograde transport) is related to an impairment of a presynaptic structural marker of RG synapses (vGlut2). Therefore, we next sought to determine the consequences of elevated IOP on RG synaptic function by performing whole-cell voltage-clamp recordings of dLGN TC neurons in a parasagittal slice preparation. First, we recorded spontaneous EPSCs (sEPSCs) in the absence of stimulation and in the presence of 60 mm picrotoxin to block GABAergic inhibition (Fig. 3). There was a statistically significant difference in sEPSC frequency between groups. While it was similar in D2-controls and 6m D2 mice, there was a significant reduction in frequency compared with controls at 9m and 12m of age. The Log_10_(sEPSC frequency) also weakly but significantly correlated with the IOP integral (Fig. 3*D*), pointing to a link between IOP and synaptic dysfunction. We found that sEPSC amplitude (Fig. 3*E*) was not significantly different between groups, suggesting that there was no detectable alteration in AMPA-receptor properties or composition at RG synapses. Although these events were recorded in the absence of tetrodotoxin (TTX), they are suitable for estimating quantal parameters in this preparation, as bath application of 500 nm TTX in a separate set of recordings had no effect on either sEPSC frequency or amplitude (*n* = 4, *p* > 0.05 paired *t* test; Fig. 3*F*), consistent with prior work in guinea pig dLGN (Paulsen and Heggelund, 1996).

**Figure 4.**
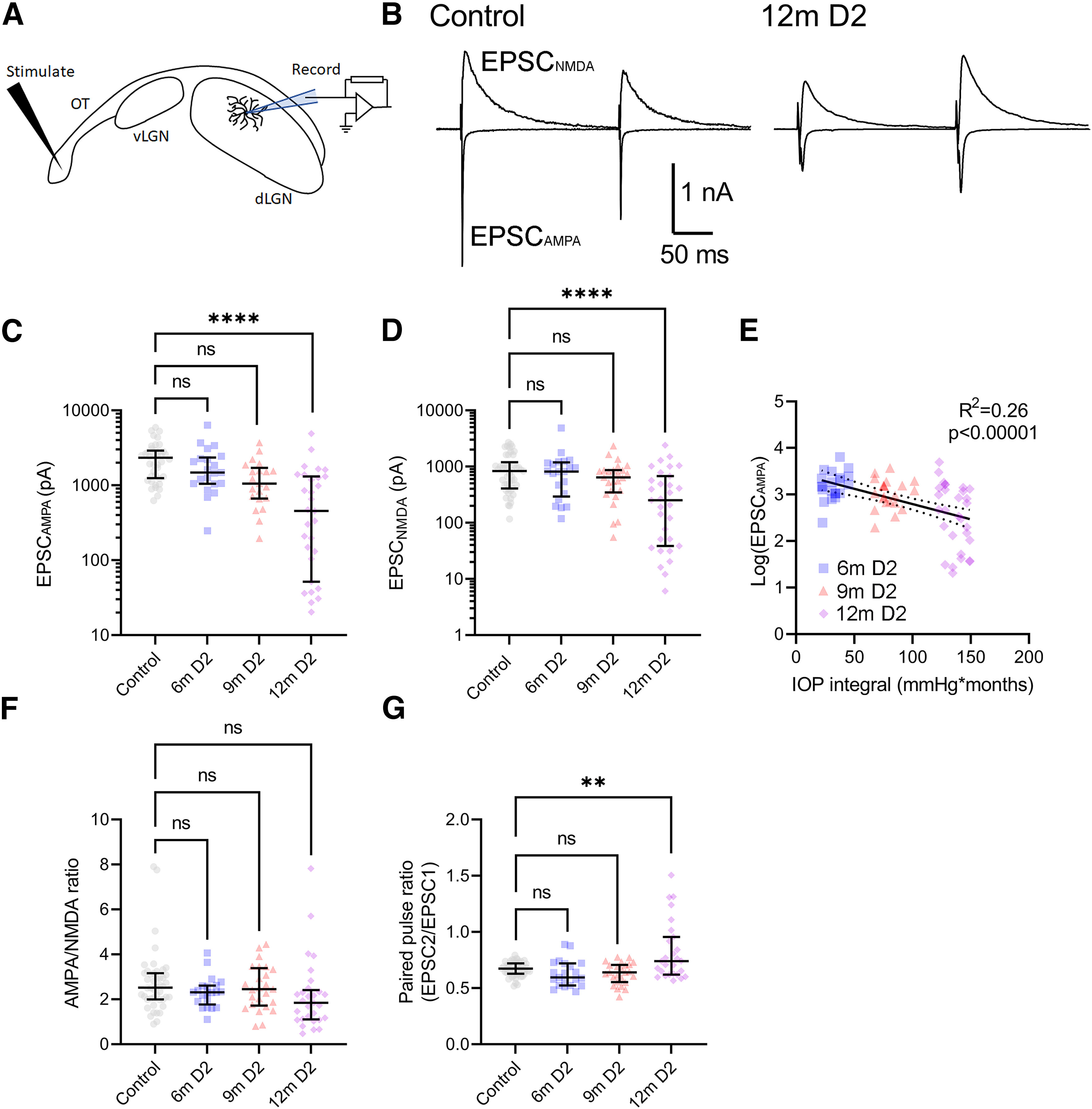
Retinogeniculate inputs to TC neurons in D2 mice. ***A***, Recording schematic of optic tract (OT) with stimulating electrode, ventral lateral geniculate nucleus (vLGN), and dLGN with patch clamp electrode in parasagittal slice. ***B***, Example maximal AMPA-receptor-mediated and NMDA-receptor-mediated EPSCs recorded at −70 and +40 mV, respectively, following maximal stimulation of the optic tract with a pair of pulses (200-ms interstimulus interval). ***C***, Group data of the AMPA-receptor-mediated maximal EPSC (EPSC_AMPA_) show that the EPSC differed among the groups (nested one-way ANOVA, *F*_(3,36)_ = 11.23, *p* = 2.4 × 10^−5^). The EPSC_AMPA_ was significantly smaller in amplitude in recordings from 12m D2 mice compared with controls (Dunnett’s multiple comparison test: 6m *p* = 0.77; 9m *p* = 0.16; 12m *p* < 1 × 10^−15^). ***D***, The NMDA-receptor-mediated EPSC differed among groups (nested one-way ANOVA, *F*_(3,38)_ = 10.51, *p* = 3.6 × 10^−5^) and the 12m amplitudes were significantly lower than control (Dunnett’s multiple comparison: 6m *p* = 0.97; 9m *p* = 0.54; 12m *p* < 1 × 10^−15^). ***E***, There was a weak but statistically significant correlation of Log_10_(EPSC_AMPA_) with the IOP integral (*F*_(1,70)_ = 24.5, *R*^2^ = 0.26, *p* = 0.0000049). ***F***, The AMPA/NMDA ratio did not significantly differ across groups (nested one-way ANOVA, *F*_(3,38)_ = 1.145, *p* = 0.34). Group sizes: control total *n* = 46 cells, 16 mice (controls by age 6m *n* = 18 cells, 5 mice; 9m *n* = 23 cells, 9 mice; 12m *n* = 5 cells, 2 mice). ***G***, Paired-pulse ratio differed among groups (nested one-way ANOVA, *F*_(3,38)_ = 6.608, *p* = 0.0011) and was significantly higher in 12m D2 mice compared with controls (Dunnett’s multiple comparison: 6m *p* = 0.75; 9m *p* = 0.79; 12m *p* = 0.0040). ***C***, ***D***, ***F***, ***G***, Median ± IQR. Sample sizes: Control, *n* = 40 cells, 12 mice (controls by age: 6m *n* = 13 cells, 4 mice; 9m *n* = 22 cells, 8 mice; 12m *n* = 5 cells, 2 mice); 6m *n* = 21 cells, 7 mice; 9m *n* = 21 cells, 9 mice; 12m *n* = 29–31 cells, 10 mice. **p < 0.01; ****p < 0.00005; ns *p* > 0.05.

A reduction in sEPSC frequency here might result from a reduction in the number of functional synapses and/or a reduction in the probability of vesicle release. To probe these possibilities, we used a stimulating electrode positioned in the optic tract to stimulate RGC axons in the presence of 60 μm picrotoxin while we recorded the maximal AMPA-receptor-mediated EPSC (EPSC_AMPA_; Fig. 4), which represents the contributions of evoked glutamate release from all intact axons converging onto a given TC neuron. In these experiments, we found that there was a statistically significant difference in EPSC_AMPA_ between groups, with a detectable reduction in EPSC_AMPA_ in slices from 12-month-old D2 mice compared with controls (Fig. 4*C*). The lower EPSC_AMPA_ amplitude was mirrored when we recorded NMDA-receptor-mediated EPSCs (EPSC_NMDA_) by changing the holding potential to +40 mV (Fig. 4*D*). There was a weak but statistically significant correlation of Log_10_(EPSC_AMPA_) with the IOP integral (Fig. 4*E*). There was no significant change in the AMPA/NMDA ratio (Fig. 4*F*), suggesting no changes in the relative contributions of each receptor type at RG synapses or “silent synapses” contributing to the reduced EPSC amplitude. When we measured the responses to a pair of stimuli delivered to the optic tract (200-ms interstimulus interval), we found that there was a statistically significant difference among the groups, with an increase in the ratio of the second response to the first [paired-pulse ratio (PPR); EPSC2/EPSC1] detectable in 12m D2 mice compared with controls (Fig. 4*G*). In some cases, the PPR was >1 in 12m D2 mice, which represents a shift from the synaptic depression more typical for RG synapses (J.P. Turner and Salt, 1998) to a mode of synaptic facilitation, suggestive of a decrease in presynaptic vesicle release probability.

**Figure 5.**
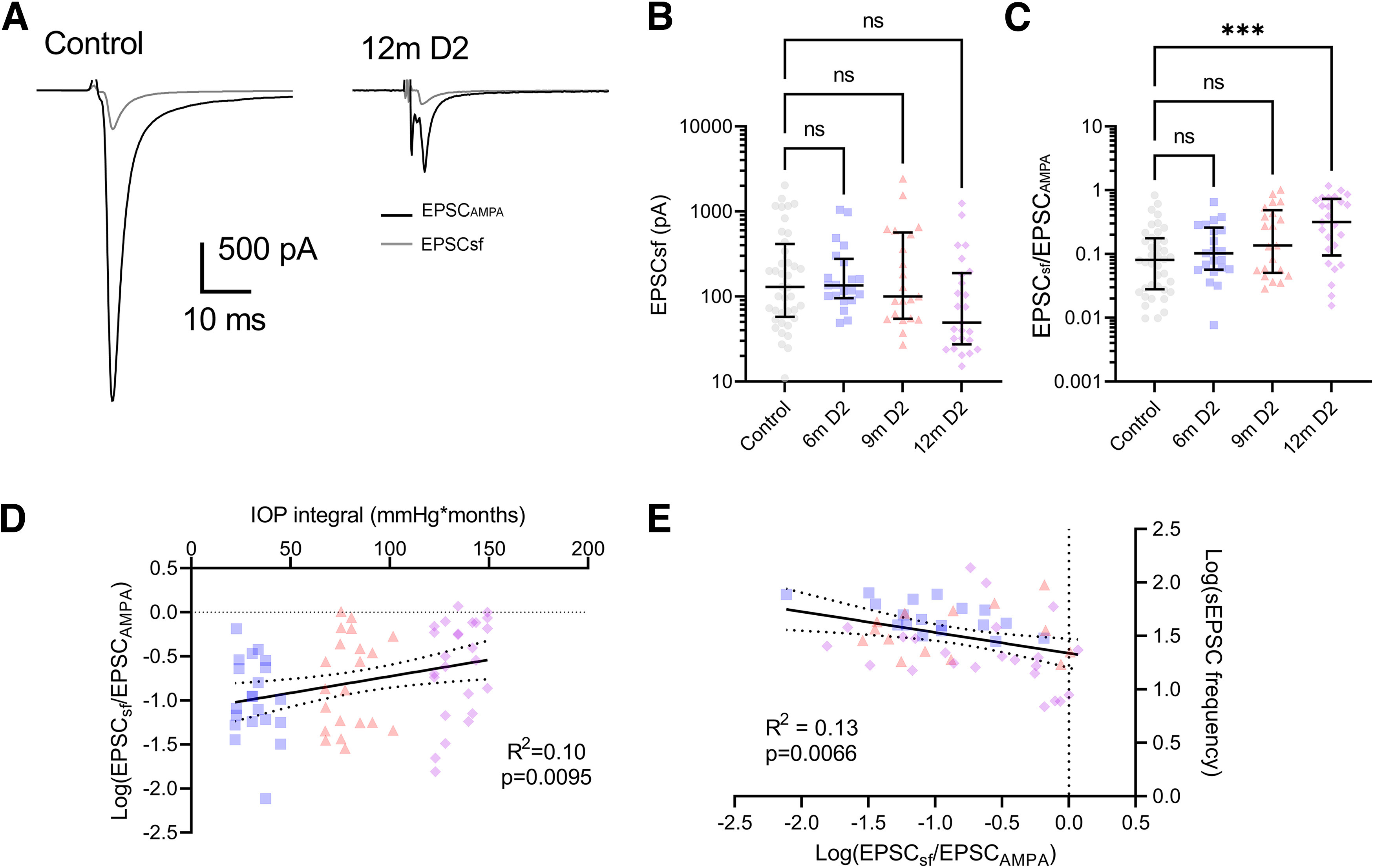
Progressive loss of convergent retinal inputs to dLGN relay neurons in DBA/2J mice. ***A***, Example maximal EPSCs (EPSC_AMPA_) and single-fiber EPSCs (EPSCsf) from a control and 12m D2 mouse. ***B***, The single-fiber EPSC amplitude (median ± IQR) did not differ among groups (nested one-way ANOVA, *F*_(3,35)_ = 1.445, *p* = 0.25). ***C***, The “fiber fraction” (EPSCsf/EPSC_AMPA_; median ± IQR) significantly differed among groups (nested one-way ANOVA, *F*_(3,35)_ = 4.604, *p* = 0.0081) and the 12m value was significantly different from control (Dunnett’s multiple comparison; 6m *p* = 0.71, 9m *p* = 0.10; 12m *p* = 0.0033). ***D***, The Log_10_(EPSCsf/EPSC_AMPA_) weakly but significantly correlated with the IOP integral in D2 mice (*F*_(1,65)_ = 7.15 *R*^2^ = 0.099, *p* = 0.0095). Sample sizes: Control, *n* = 40 cells, 12 mice (controls by age: 6m *n* = 13 cells, 4 mice; 9m *n* = 22 cells, 8 mice; 12m *n* = 5 cells, 2 mice); 6m *n* = 21 cells, 7 mice; 9m *n* = 21 cells, 9 mice; 12m *n* = 26 cells, 9 mice. ***E***, Log_10_(sEPSC frequency) weakly but significantly correlated with Log_10_(EPSCsf/EPSC_AMPA_; *F*_(1,53)_ = 7.99, *R*^2^ = 0.13, *p* = 0.0066). 6m *n* = 18 cells, 7 mice; 9m *n* = 14 cells, 7 mice; 12m *n* = 23 cells, 9 mice. ***p < 0.005; ns *p* > 0.05.

We next reduced the stimulus amplitude to evoke synaptic vesicle release from single RGC axons (Fig. 5). The ratio of the single fiber EPSC to the maximal EPSC (EPSCsf/EPSC_AMPA_; the “fiber fraction”) has been used to monitor the developmental refinement of RGC inputs onto TC neurons and represents a statistically quantifiable estimate of synaptic convergence (Hooks and Chen, 2006, 2008; Litvina and Chen, 2017a). Although there was no significant effect on EPSCsf (Fig. 5*B*), we found that EPSCsf/EPSC_AMPA_ was significantly increased in 12m D2 mice compared with controls (Fig. 5*C*), indicating a reduction in the number of functional RGC axon inputs onto each TC neuron without substantive change in the contribution from individual RGC axons. The Log_10_(EPSCsf/EPSC_AMPA_) was weakly, but significantly, correlated with the IOP integral, consistent with a link between eye pressure and loss of RG synapse function in D2 mice (Fig. 5*D*).

Taking the reciprocal of the fiber fraction suggests that each TC neuron receives inputs from an average 6.9 RGC axons in control mice, while 12m D2 mice receive an average of 2.3 functional RGC axon inputs. At the same time point, we detected a reduction in sEPSC frequency by 18 Hz; control sEPSC frequency was 37 Hz, while it was 19 Hz in 12m D2 mice. Comparison with published anatomic studies of RG synapses points to the congruence of these two measurements (Morgan et al., 2016; Litvina and Chen, 2017b); if each RGC axon contributes 15 boutons to a postsynaptic TC neuron, each with 27 active zones having a vesicle fusion rate of 0.01 Hz per active zone in the absence of stimulation (Murthy and Stevens, 1999), then the expected result of a drop from 6.9 RGC axonal inputs to 2.3 inputs is an 18-Hz reduction in event frequency. Moreover, for the subset of TC neurons in which we measured both sEPSC frequency and EPSCsf/EPSC_AMPA_, there was a congruence of these two measurements; Log_10_(sEPSC frequency) was significantly correlated with Log_10_(EPSCsf/EPSC_AMPA_; Fig. 5*E*). This indicates that the change in synaptic transmission measured with both quantal events and optic tract stimulation are of a similar scale and likely to represent complementary measures of a similar pathologic process.

**Figure 6.**
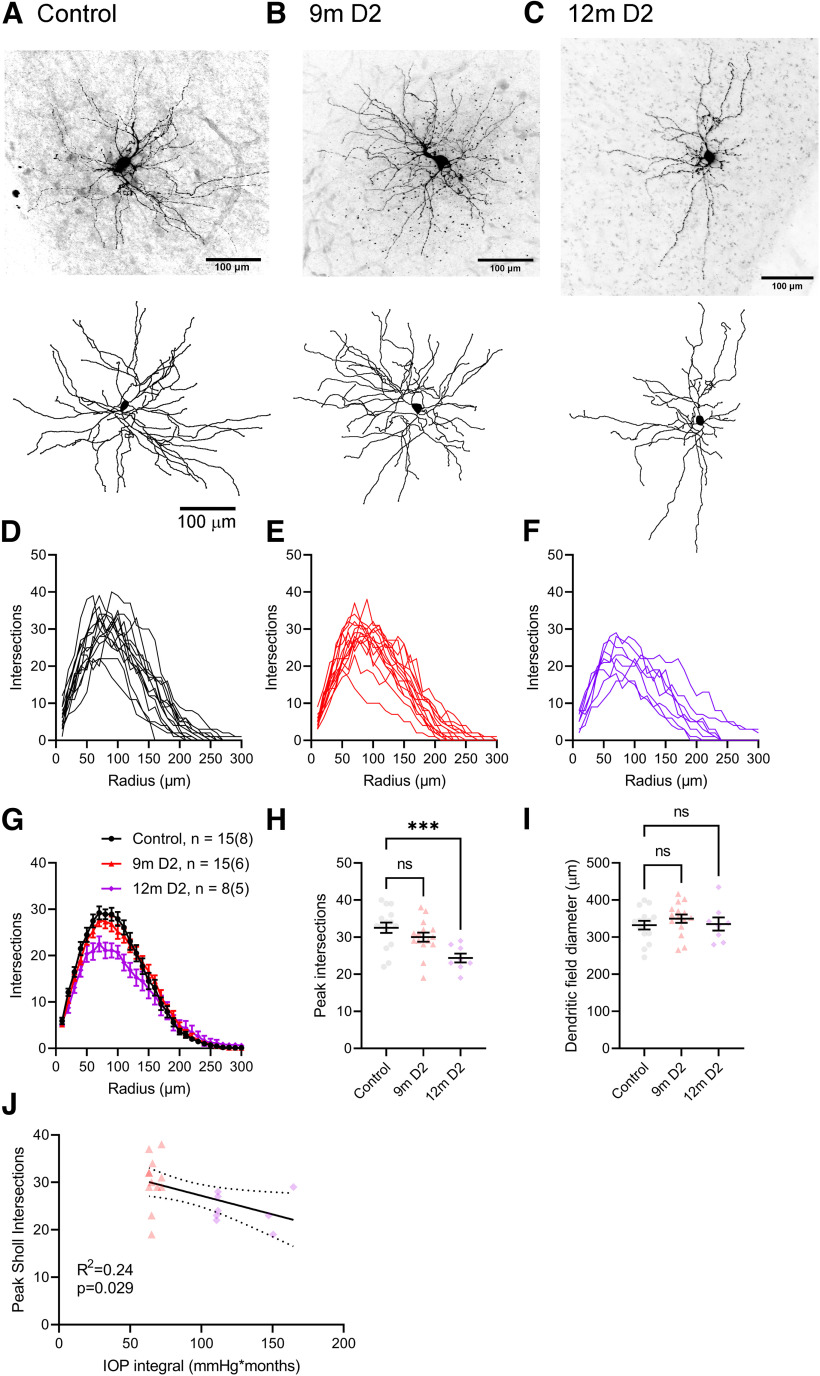
Thalamocortical neuron dendritic remodeling in DBA/2J mice. ***A–C***, Top row, Maximum intensity projections of filled TC neurons filled with Neurobiotin during whole-cell recording in coronal slices from control (***A***), 9m D2 (***B***), and 12m D2 (***C***) mice. Bottom row, TC neuron dendrite reconstructions. ***D–F*,** Sholl plots of each TC neuron included in the sample. ***G***, Group data (mean ± SEM) of Sholl plots. ***H***, Group data (mean ± SEM) of the peak number of Sholl intersections for each cell. There was a significant difference among groups (nested one-way ANOVA, *F*_(2,16)_ = 8.346, *p* = 0.0033) and the 12m peak intersections was significantly lower than control (Dunnett’s multiple comparison, 9m *p* = 0.46; 12m *p* = 0.0019). ***I***, Group data (mean ± SEM) of the dendritic field diameter measured as the equivalent diameter of a convex polygon of the dendritic field. There was no statistically significant difference among groups (nested one-way ANOVA, *F*_(2,16)_ = 0.2320, *p* = 0.80). ***J***, There was a weak but statistically significant correlation of the peak number of Sholl intersections with the IOP integral for mice with complete IOP records (*F*_(1,18)_ = 5.65, *R*^2^ = 0.24, *p* = 0.029). Sample size: Control *n* = 15 cells (8 D2-control mice ages 4m–12m); 9m–10m D2 *n* = 15 cells (7 mice); 12m D2, *n* = 8 cells (5 mice). ***p < 0.005; ns *p* > 0.05.

The above findings show a loss of vGlut2-labeling of RGC axon terminals and diminished numbers of RGC inputs to each TC neuron in older DBA/2J mice. However, postsynaptic TC neuron structure and function are likely to be altered in glaucoma as well. For instance, elevated IOP is associated with altered TC neuron intrinsic excitability and somatic atrophy (Van Hook et al., 2020). Prior studies have also reported reorganization of LGN neuron dendrites in late-stage glaucoma in primates (Gupta et al., 2007; M. Liu et al., 2011; Ly et al., 2011), and we have previously shown changes in TC dendritic structure following bilateral enucleation (Bhandari et al., 2022), a traumatic form of optic nerve injury, and IOP elevation with microbead injections (Bhandari et al., 2019). Here, we performed Sholl analysis of TC neuron dendrites reconstructed after neurobiotin filling during whole-cell recording and found that although TC neuron dendritic complexity was comparable between control and 9m D2 mice, there was a modest reduction in the peak number of Sholl intersections in 12m D2 mice compared with controls (Fig. 6). There was no statistically significant difference in equivalent dendritic field diameter among the groups. We also detected a weak but statistically significant correlation of the peak number of Sholl intersections with IOP integral (Fig. 6*J*). Thus, reorganization of postsynaptic TC neuron structure accompanies loss of function of RG synaptic inputs, and this is related to IOP.

**Figure 7.**
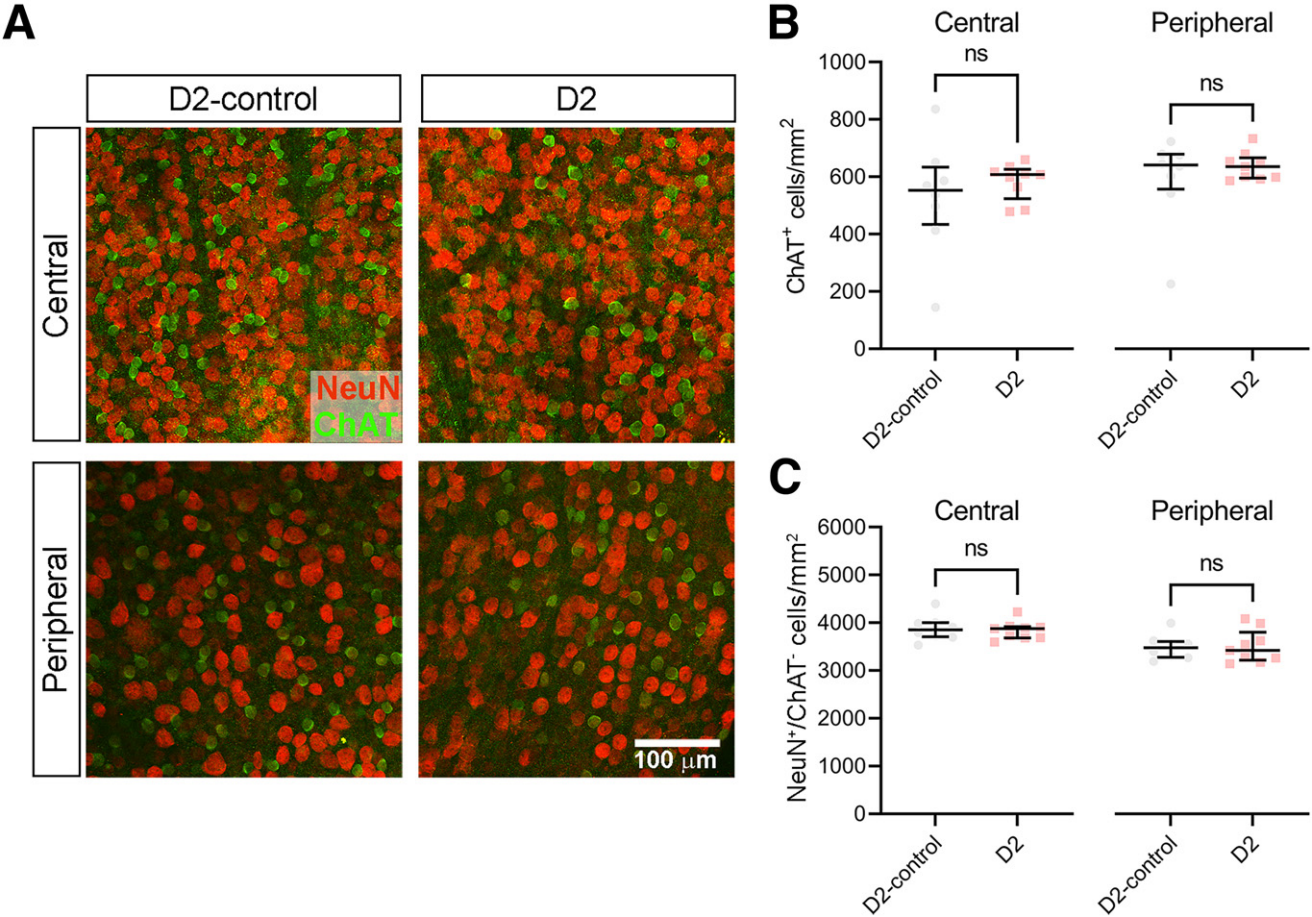
No loss of retinal ganglion cell somata in 11- to 12-month-old DBA/2J mice. ***A***, Two-photon immunofluorescence images of retinal flat mounts from a 12-month-old D2-control mouse and a 12-month-old D2 mouse stained with antibodies for NeuN and choline acetyltransferase (ChAT). Images were acquired from central retina (centered ∼500 μm from the optic nerve head) and peripheral retina (centered ∼1700 μm from the optic nerve head). ***B***, Analysis of ChAT^+^ cell density (median ± IQR). Each data point is the ChAT^+^ cell density averaged across three to four quadrants for each retina. There was no significant difference in ChAT^+^ cell density between D2-control and D2 mice (central *t*_(15)_ = 0.7860, *p* = 0.44; peripheral *t*_(15)_ = 0.8289, *p* = 0.42, unpaired *t* test). ***C***, RGC density was measured as the difference between the total number of NeuN^+^ cells and the number of NeuN^+^/ChAT^+^ double-labeled cells. There was no significant difference between D2-control and D2 RGC density (central *t*_(15)_ = 0.4230, *p* = 0.68; peripheral *t*_(15)_ = 0.0920, *p* = 0.93, unpaired *t* test). Sample sizes: D2-control, *n* = 8 retinas, 4 mice; D2, *n* = 9 retinas, 5 mice. ns *p* > 0.05.

We next sought to count the number of RGC somata in D2 retinas to provide another comparison of the above findings with a commonly used metric of glaucomatous progression (Fig. 7). The use of the neuronal marker NeuN, in conjunction with correction of counts for NeuN-positive cholinergic amacrine cells in the ganglion cell layer (identified by labeling for choline acetyltransferase; ChAT) is a reliable way of counting RGCs and has been used previously to show that somatic loss is a late event in DBA/2J mice (Buckingham et al., 2008; Calkins, 2021). We found that the density of ChAT^+^ cells in the RGC layer was not significantly different between D2 and D2-control mice (11–12 months age), although it was slightly lower than previously reported in C57Bl/6J and A/J mice, which likely reflects strain differences (Keeley et al., 2007; Whitney et al., 2008). We measured the density of NeuN^+^ presumptive RGCs by correcting for ChAT^+^/NeuN^+^ double-labeled cells, finding that there was no significant difference in the density of NeuN^+^ RGCs. This is consistent with and provides confirmation of prior work suggesting that RGC somatic loss is a late event in DBA/2J mice (Buckingham et al., 2008).

## Discussion

This study demonstrates a loss of RGC output synapses in the dLGN in a mouse model of glaucoma occurring before degeneration of RGC somata. This involves the drop-off of individual RGC inputs to postsynaptic TC neurons without appreciable alterations in the strength of individual RGC inputs. We also find that IOP elevation is associated with diminished anterograde optic tract transport to the dLGN and loss of vGlut2-labeled RGC terminals. These presynaptic effects were followed by a modest loss of dendritic complexity in proximal regions of TC neuron dendritic arbors, which might represent a disruption of dendritic homeostasis because of diminished retinogeniculate synaptic inputs. While some results of the current study parallel findings in the superior colliculus in DBA/2J mice and other inducible models of glaucoma (Crish et al., 2010; H. Chen et al., 2015; Smith et al., 2016), our use of patch clamp electrophysiology studies to probe the functional loss of RGC output synapses in glaucoma is unique and highlights the functional decline of RGC outputs. Although the rodent dLGN receives comparatively fewer RGC inputs than the colliculus, it is an important retinal projection target for conscious vision (Martin, 1986; Ellis et al., 2016; Seabrook et al., 2017). Likewise, its primate equivalent, the LGN, is central to visual function for humans and nonhuman primates. The rodent dLGN is therefore an approachable system for understanding the impact of eye pressure and glaucomatous degeneration on central visual system function with key relevance for human glaucoma patients (Gupta et al., 2006, 2009).

We used two complementary electrophysiological analyses of dLGN synapses: (1) measurements of sEPSC frequency and (2) evoked RG transmission and fiber fraction measurements. The use of fiber fraction measurements (Hooks and Chen, 2006, 2008; Litvina and Chen, 2017a) showed that TC neurons from 12m D2 mice receive inputs from fewer RGCs than in controls. While the sEPSC results are consistent with several scenarios including loss of synaptic contacts made by each RGC axon or weakening of individual inputs, our data obtained with optic tract stimulation do not indicate a change in the number of bouton contacts or a major change in the strength of individual synapses, as either process would be reflected in a reduction in the EPSCsf. This instead largely results from drop-off of individual RGC axons, as evidenced by the increase in fiber fraction and no statistically detectable change in single fiber strength. We also observed a change in short-term plasticity, with an increase in the paired-pulse ratio of RG synapses in 12m D2 mice, with some even displaying paired pulse facilitation in contrast to the paired pulse depression typical at these synapses. This change in short-term plasticity likely reflects a reduction in presynaptic vesicle release probability, the origins of which might lie in altered presynaptic calcium dynamics because of compromised mitochondrial health (Smith et al., 2016). Clarifying this will require further study. Notably, using the microbead approach to raise IOP, we have previously found an increase in retinogeniculate vesicle release probability, although we did see a decrease in sEPSC frequency and a similar reduction in TC neuron dendritic complexity (Bhandari et al., 2019), more in line with the current study. The differential effects on release probability are interesting and might reflect differences in mouse age (we used younger mice in the prior study), as older animals have been shown to display more pronounced pathology in response to experimentally-elevated IOP (Crish et al., 2010). Alternatively, the extent and duration of the IOP increase might account for some of the differences, as the microbead model evoked a very modest IOP increase and *ex vivo* experiments were performed only five weeks postbead injection.

vGlut2 labeling studies showed a progressive vGlut2 loss from 9m to 12m. We found that vGlut2 density was related to CTb transport integrity, although a considerable amount of vGlut2 was still present in regions with minimal CTb, suggesting that loss of RGC axon terminal labeling lags transport deficits in the dLGN, although other sources of vGlut2 terminals such as the vGlut2-positive terminals arising from superior colliculus might contribute (Fremeau et al., 2001). It is unknown whether the diminished dLGN vGlut2 labeling in D2 mice reflects degeneration of RGC axon terminals or loss of vGlut2 protein, perhaps because of perturbed transport from the soma. Ultrastructural studies of the D2 SC show that RGC axon terminals persist in regions deficient for transported CTb (Crish et al., 2010) although they are atrophied and have misshapen mitochondria and smaller active zones (Smith et al., 2016). We did not detect any statistically significant differences in vGlut2 punctum size between transport-intact and -deficient regions at the level of light microscopy. While this might represent a contrast with the RGC axon terminal pathology in the SC (Smith et al., 2016), future ultrastructural studies along with measurements of mitochondrial function will be necessary to test this in the dLGN. We also found that CTb transport to the dLGN was weakly correlated with IOP. Notably, this differs from work in SC, where no such correlation was found (Crish et al., 2010). This might stem from different susceptibility to transport deficits in RGC subpopulations projecting to SC versus dLGN or might instead be reflective of different sensitivity in each study to detect the weak correlation.

In addition to the presynaptic deficits (vGlut2 labeling, CTb transport, and electrophysiological measures), we also show that TC neurons in D2 mice display reorganization of their postsynaptic dendrites. Such dendritic reorganization is a common feature of neurodegenerative diseases (Y.C. Lin and Koleske, 2010). Prior evidence from primate LGN has identified some dendritic loss in glaucoma (Gupta et al., 2007; M. Liu et al., 2011; Ly et al., 2011) and we have previously found reductions in TC neuron dendrites in microbead-induced ocular hypertension and following enucleation (Bhandari et al., 2019, 2022). Here, in 12m D2 mice, we find reduced TC neuron dendritic complexity proximal to the soma. This region has a higher concentration of RG inputs compared with the distal dendrites (Robson, 1993; Morgan et al., 2016), where there is a greater concentration of weak corticothalamic feedback synapses. Synaptic inputs are important for dendritic maintenance, with deafferentation or reduced synaptic strength being salient triggers for dendritic loss (Deitch and Rubel, 1989; Flood and Coleman, 1993; Y.C. Lin and Koleske, 2010). This is a potentially important role of spontaneous synaptic transmission, with spontaneous synaptic input serving a homeostatic function for synaptic maintenance. Retinal input is important for TC neuron dendritic development (El-Danaf et al., 2015; Charalambakis et al., 2019). Thus, it is likely that dendritic loss here is primarily a response to rather than a cause of diminished retinogeniculate synaptic strength. It remains to be tested, however, whether the loss of postsynaptic dendritic complexity is preceded by loss of postsynaptic contacts (i.e., PSD-95 puncta) in D2 TC neurons. Alternatively, portions of the remaining dendritic arbor might lack functional synaptic contacts. This possibility should be explored in future studies. Prior work has also documented several morphologic cell classes in mouse dLGN (W-cells, X-cells, and Y-cells) with preference for different dLGN regions (Krahe et al., 2011). Our sample focused on the dLGN core region, which is populated mostly by cells with a Y-type radial morphology in healthy animals. It is possible that pathology can complicate identification of cell type by changing dendritic morphology. Additionally, it is unclear whether each cell type will respond to glaucomatous pathology in a similar fashion and this possibility remains to be tested.

What is the relationship between dLGN synaptic function and RGC somatic degeneration? Vision impairment in glaucoma is sometimes linked with RGC apoptosis, although numerous degenerative events in somatic/dendritic, axon, and axon terminal “compartments” precede detectable somatic loss (Calkins, 2021). We found no detectable RGC loss in 11- to 12-month-old D2 mice, which is consistent with prior work showing that RGC somatic loss in D2 mice does not occur until after 15 months of age (Buckingham et al., 2008). RGCs do undergo numerous other structural and functional changes before somatic loss in D2 mice including altered dendritic complexity and synapse loss (Della Santina et al., 2013; Williams et al., 2013; Agostinone and Di Polo, 2015; Ou et al., 2016; Risner et al., 2018; Bhandari et al., 2022), intrinsic excitability (Della Santina et al., 2013; Ou et al., 2016; Risner et al., 2018), light responses (Della Santina et al., 2013; Ou et al., 2016; Risner et al., 2018), and metabolic function (Casson et al., 2021; H. Liu and Prokosch, 2021). Optic nerve pathology including axon expansion, axon loss, reduced neurofilament staining, and astrocyte reorganization are associated with age and IOP and appear to precede RGC somatic loss in DBA/2J mice (Inman et al., 2006; Buckingham et al., 2008; Cooper et al., 2016). Thus, the results of the current study support the body of evidence indicating that pathologic changes to visual system structure and function before RGC somatic loss, in this case, diminishment of visual information transfer at the retinogeniculate synapse, contribute to visual impairment in glaucoma.

There are several limitations with the current study and areas for future exploration. First, as discussed above, our results do not differentiate whether glaucoma leads to degenerative loss of synapses, as loss of vGlut2 labeling might result from deficits in axon transport. Moreover, while we show that TC neurons lose dendritic complexity in 12m D2 mice, we do not know whether this is accompanied by a concurrent loss of postsynaptic markers (such as PSD95). Ultrastructural studies of presynaptic and postsynaptic contacts will be needed to ascertain whether these structures remain intact. Second, differences in dLGN regions receiving input from RGC axons with intact versus deficient CTb transport might contribute to the variability in measurements of synaptic function we show here. Our data show a relationship between CTb transport integrity and vGlut2 and prior work has shown that transport deficits are related to ultrastructural defects in presynaptic RGC axon terminals in the SC (Crish et al., 2010; Dengler-Crish et al., 2014; Smith et al., 2016). We have not yet explored the link between transport integrity and synaptic function in the dLGN. Third, while we have shown previously that TC neurons are more excitable D2 mice (Van Hook et al., 2020), which might represent a homeostatic maintenance of thalamocortical information transfer following diminished RG synapses, we have not yet explored the consequences of these two phenomena operating in concert; enhanced TC neuron excitability might maintain signaling to the visual cortex until a tipping point in the disease process after which the fidelity of visual signaling is impaired.

## Synthesis

Reviewing Editor: Matthew Grubb, King’s College London Decisions are customarily a result of the Reviewing Editor and the peer reviewers coming together and discussing their recommendations until a consensus is reached. When revisions are invited, a fact-based synthesis statement explaining their decision and outlining what is needed to prepare a revision will be listed below. The following reviewer(s) agreed to reveal their identity: Samuel Crish. Note: If this manuscript was transferred from JNeurosci and a decision was made to accept the manuscript without peer review, a brief statement to this effect will instead be what is listed below.

Reviewer 1:

This is an elegant paper investigating the retinogeniculate projection in a common mouse model of glaucoma. Using a combination of electrophysiological and histological methods, the authors do a great job interrogating the retinal projection to the LGN, the major relay station for visual information on the way to the cortex. This is a substantial contribution to the literature, especially considering the importance of this structure in humans - much of the mouse glaucoma research focuses on the superior colliculus. This is great neuroscience - the field needs more papers like this that look at both the anatomy and physiology of the retinal projection.

I believe the authors have adequately addressed the previous reviewers’ comments and I have only minor suggestions for consideration.

1) The LGN is by no means a minor retinal target. I think the LGN is underresearched in mouse glaucoma. Given the direction the field is going in, re: vulnerabilities of different RGCs, etc., (which the authors do a good job discussing) looking at the different visual centers in the brain provides valuable information. I’d like to encourage the authors to soften their softening of the importance of the LGN.

2) The authors found a correlation between transport deficits and IOP that similar studies did not find. Can the authors comment on why this is different, or if they even think it is different? i.e., perhaps since the correlation is weak in this model and the previous studies weren’t sensitive enough to detect it?

3) Line 279: I’m not sure the authors are testing the influence of IOP on axon terminals, given the complexity of IOP’s role in pathology in DBA/2J mice.

4) Line 439: I’m not following the logic here. I don’t see how the other statements in this paragraph would lead to the conclusion that terminals remained after VGluT2 immunopositivity. Wouldn’t the slow turnover of synaptic proteins and presence of VGluT2 after transport loss suggest that VGluT2 is a good marker of terminal persistence, rather than the terminals persist after VGluT2 loss? Please clarify if I am misreading it.

5) Though beta is used in some manuscripts, it’s actually Cholera Toxin B subunit, not beta.

Reviewer 2:

This nicely done study examines the retinogeniculate synapse in the DBA/2J mouse model of experimental glaucoma. Examining both structure and function, the authors find that there is early-stage loss and dysfunction of RGC output synapses in the dLGN and that treatment with brimonidine prevented loss of RGC synaptic labeling.

The authors have done a commendable job of responding to their prior reviewers’ comments, and this reviewer had similar questions that were already answered (correlations between Neuobiotin filled cells’ structure and function, which unfortunately was not collected, cumulative IOP vs age issues, etc). Some additional issues remain:

1. With regard to cumulative IOP, I found the authors’ response to the first set of reviews puzzling. There are correlations plotted in the various figures, but sometimes 2 month cumulative IOP is presented, whilst sometimes 3 month cumulative IOP is presented. Furthermore, cumulative IOP appears just to be the sum of the IOPs. To accurately report the IOP integral, it should be reported as an AUC with the units as mmHg * days, and should include all IOP measurements (not just the preceding 2-3 months, given the heterogeneity of IOP in these animals). Figure 2 should (like the other figures) report the relationship between the IOP integral and vGLuT2 density. The IOP integral should be reported in all figures where IOP is reported, including the brimonidine experiment.

2. The Discussion would benefit from a brief discussion of the findings in this work compared to the authors’ own previously published work using an inducible model, especially given the differences in structural and functional measures identified 5 weeks after microbead injection compared to this study.

3. Do the statistics account for paired eyes, for example in the brimonidine experiment as both eyes are used in the experiment (i.e. n = 18 eyes, 9 mice, was each eye treated as independent)?

4. The brimonidine experiment is not very informative (in the sense that its IOP lowering and neuroprotective effects are well-established), and the authors should consider removing it. We do not know from the study whether brimonidine actually rescues function at the retinogeniculate synapse, and the reader is left hanging.

Other comments:

1. Were the analyses done in a masked fashion?

2. The authors should re-label the NeuN/ChAT data in the corresponding Figure and in the text to indicate that they are counting the population of NeuN positive ChAT negative cells. I am not sure why they chose to go this route for RGC counts rather than using an established marker such as RBPMS. Yes, it’s true that these markers can be downregulated, but that is true for NeuN as well. Furthermore, the authors note that only 66% ChAT positive cells were NeuN positive, so their method has its own issues for “RGC” counting.

3. Please provide raw images of the cell fills for Figure 6.
